# Production of the antifungal biopesticide physcion through the combination of microbial fermentation and chemical post-treatment

**DOI:** 10.1186/s40643-023-00625-8

**Published:** 2023-01-09

**Authors:** Zheng Zhuang, Xueqing Zhong, Qinghua Li, Tian Liu, Qing Yang, Guo-Qiang Lin, Qing-Li He, Qunfei Zhao, Wen Liu

**Affiliations:** 1 The Research Center of Chiral Drugs, Innovation Research Institute of Traditional Chinese Medicine, Shanghai University of Traditional Chinese Medicine, 1200 Cailun Road, Shanghai, 201203 China; 2 State Key Laboratory for Biology of Plant Diseases and Insect Pests, Institute of Plant Protection, Chinese Academy of Agricultural Sciences, No. 2 West Yuanmingyuan Road, Beijing, 100193 China; 3 State Key Laboratory of Bioorganic and Natural Products Chemistry, Shanghai Institute of Organic Chemistry, Chinese Academy of Sciences, 345 Lingling Road, Shanghai, 200032 China; 4 School of Bioengineering, Dalian University of Technology, No. 2, Linggong Road, Dalian, 116024 China

**Keywords:** Antifungal biopesticide, Physcion, Microbial production

## Abstract

Physcion is an anthraquinone compound observed dominantly in medicinal herbs. This anthraquinone possesses a variety of pharmaceutically important activities and has been developed to be a widely used antifungal biopesticide. Herein, we report on the effective preparation of *3R*-torosachrysone (**4**), a tetrahydroanthracene precursor of physcion, in *Aspergillus oryzae* NSAR1 by heterologous expression of related genes mined from the phlegmacins-producing ascomycete *Talaromyces* sp. F08Z-0631. Conditions for converting **4** into physcion were studied and optimized, leading to the development of a concise approach for extracting high-purity physcion from the alkali-treated fermentation broth of the **4**-producing *A. oryzae* strain.

**Graphical Abstract:**

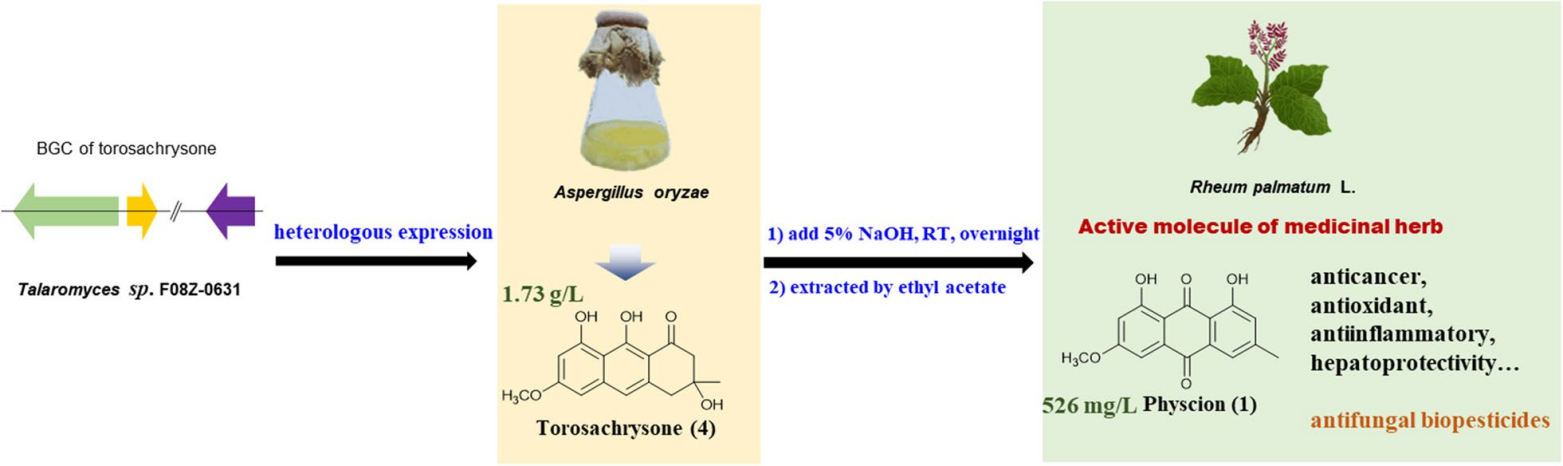

**Supplementary Information:**

The online version contains supplementary material available at 10.1186/s40643-023-00625-8.

## Introduction

**Fig. 1:**
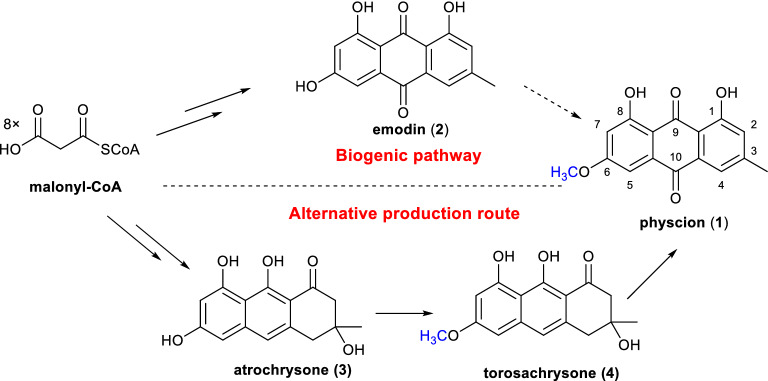
Putative biogenic pathway of anthraquinone fungicide physcion and the alternative production route developed in this paper

Anthraquinone physcion (**1**), 1,8-dihydroxy-6-methoxy-3-methylanthracene-9,10-dione (Fig. 1), is abundantly distributed in *Polygonaceae* family plants, including the traditional Chinese medicinal herbs *Rheum palmatum* L., *Polygonum multiflorum* (Thunb.), and *Polygonum cuspidatum*. It has displayed anticancer, antioxidant, anti-inflammatory, antibacterial, antiviral and hepatoprotective activities (Ji et al. 2021; Xunli et al. 2019). Notably, it has already been commercially available as a low-toxicity and eco-friendly biological fungicide used to prevent and treat powdery mildew in agriculture in China (Li et al. 2022; Yang et al. 2007, 2008).

In industrial scalable preparation, physcion (**1**) is mostly extracted from the roots and stems of *R. palmatum* L. and then, obtained with conventional phytochemical separation techniques (Li et al. 2022). The average yield of rhubarb is 300 kg/mu (1 mu = 667 m^2^), while the concentration of physcion is only 0.01–0.2% (Qi et al. 2022). Therefore, it requires considerable time and labour to plant and harvest, and its productivity is constrained by plant resources. Moreover, because a variety of anthraquinone compounds with similar physico-chemical properties, such as emodin (**2**), chrysophanol, and aloe emodin, frequently exist in the same plants, physcion and its analogues are so poorly soluble in water that obtaining high-purity physcion is detrimental to resources and the environment due to the high solvent consumption and prolonged extraction processes (Wang et al. 2013). In addition to plant sources, physcion was also found among the metabolites of several terrestrial and marine fungi, albeit often with very low fermentation yields. For instance, the yields of physcion in the marine fungus *Microsporum* sp. (Wijesekara et al. 2014) and *Aspergillus parasiticus* (Anderson 1986) were as low as 0.59 mg/L and 1.2 mg/L, respectively.

Microbial fermentation by constructing cell factories through synthetic biology is a promising way of producing important plant-derived natural products efficiently and sustainably. The complete microbial manufacture of numerous herbal natural product compounds with significant therapeutic potential, such as vinblastine (Caputi et al. 2018), colchicine (Nett et al. 2020), scopolamine (Srinivasan et al. 2020), and strychnine(Hong et al. 2022), has been effectively accomplished in recent years thanks to the rapid development of synthetic biology technology. Understanding the target molecule’s biosynthetic pathway and the mechanism of key enzyme-catalyzed reactions is essential for the effective use of synthetic biology. Physcion (**1**) has long been considered as a C6 *O*-methylation derivative of emodin (**2**), which is the common intermediate of a very large group of secondary metabolites, including anthraquinone chrysophanol and cladofulvin, benzophenone monodictyphenone, and xanthone ergochrome (De Mattos-Shipley et al. 2022) (Fig. 1). It has been shown that the putative precursor emodin **2** is formed by an octaketide synthase (OKS), a type III polyketide synthase (PKS) superfamily member, *in planta* (Guo et al. 2021; Karppinen et al. 2008; Abe et al. 2005; Mizuuchi et al. 2009)*,* and by a nonreducing iterative type I PKS (NR-iPKS, also known as atrochrysone carboxylic acid synthase, ACAS), in conjunction with a physically discrete metallo-β-lactamase-type thioesterase (MβTE, also named atrochrysone carboxyl ACP thioesterase, ACTE) in fungi (Awakawa et al. 2009; Griffiths et al. 2016; Han et al. 2021). However, the biosynthetic pathway of emodin has not been fully completely elucidated, particularly in plants. As early as 1986, Anderson J.A. found that the cell-free extract of the filamentous fungus *Aspergillus parasiticus,* which produces physcion (**1**), catalyzed the conversion of [^3^H] **2** and *S*-adenosyl-l-[Me-^14^C]methionine to ^3^H- and ^14^C-labelled **1** (Anderson 1986). The enzymes that can specifically catalyze the C6–OH methylation of **2** have not been identified, although a methyltransferase that catalyzes the C8–OH methylation of **2** to form emodin-8-methyl ether (also known as questin) from *Aspergillus terreus* has been cloned and characterized (Chen et al. 1992; Xue et al. 2022). Very recently, by screening methyltransferase from different aromatic compound biosynthetic pathways, Qi, F. et al. obtained an enzyme that can catalyze C6–OH methylation of emodin to form physcion. They then constructed a physcion-producing strain by integrating this enzyme into an engineered *A. terreus* chassis strain that accumulates emodin (Qi et al. 2022).

Herein, in the absence of a completely understood biosynthetic pathway, we developed an alternate production route for physcion (Fig. 1). We constructed a high-yield *3R*-torosachrysone (**4**)-producing *A. oryzae* strain and explored the method of transforming compound **4** into physcion. Subsequently, we efficiently obtained physcion by the combination of microbial fermentation and chemical post-treatment. This method provides an alternative method for producing plant-derived antifungal pesticide physcion in an effective, sustainable and environmentally friendly manner. Our approach will also encourage the microbial synthesis of more plant-derived compounds, whose biosynthetic pathways are more challenging to elucidate.

## Materials and methods

### General materials and methods

Biochemicals and media were purchased from Sinopharm Chemical Reagent Co., Ltd. (China), Shanghai Sangon Biotech Co. Ltd. (China), Oxoid Ltd. (U.K.) or Sigma‒Aldrich Corporation (USA) unless otherwise stated. Restriction endonucleases were purchased from New England Biolabs, Inc. Ltd. (USA). Chemical reagents were purchased from standard commercial sources. The primers, plasmids and strains used in this study are summarized in Additional file 1: Tables S1–S3.

Primer synthesis and DNA sequencing were performed at Shanghai Sangon Biotech Co. Ltd. (China). Plasmid purification kits and agarose gel DNA extraction kits were purchased from Omega Biotek (USA). PCR amplifications were carried out on an Applied Biosystems Veriti^™^ Thermal Cycler using either Taq DNA polymerase (Vazyme Biotech Co. Ltd, China) for routine genotype verification or Phanta^®^ Max Super-Fidelity DNA Polymerase (Vazyme Biotech Co. Ltd, China) for high-fidelity amplification. ClonExpress^®^ One Step Cloning Kits were purchased from Vazyme Biotech Co., Ltd. China. Transcriptome sequencing of *Talaromyces sp.* F08Z-0631 was performed at Novogene Co., Ltd. (China).

High-performance liquid chromatography (HPLC) analysis was conducted with Agilent 1260 Infinity II HPLC systems (Agilent Technologies Inc., USA). Electrospray ionization mass spectrometry (ESI–MS) was performed on an AB Sciex QTRAP^®^ 6500^+^ mass spectrometer (AB Sciex Pte. Ltd., USA), and the data were analyzed using SCIEX OS Software. High resolution–ESI–MS (HR–ESI–MS) analysis was realized with an Agilent 6545 Accurate-Mass QTOF LC/MS System (Agilent Technologies Inc., USA), and the data were analyzed using Agilent MassHunter Qualitative Analysis software. Nuclear magnetic resonance (NMR) data were recorded on Bruker AVANCE NEO 600 M spectrometers (Bruker Co. Ltd, Germany).

### Sequence analysis

Biosynthetic gene clusters were predicted with the fungal version of antiSMASH (https://fungismash.secondarymetabolites.org/). The deduced proteins were compared to other known proteins in databases using the available BLAST methods (http://blast.ncbi.nlm.nih.gov/Blast.cgi). Multiple sequence alignments and the phylogenetic tree were created using Clustal Omega (https://www.ebi.ac.uk/Tools/msa/clustalo/) with default settings. The visualization of sequence alignments was created using ESPript 3.0 (https://espript.ibcp.fr/ESPript/ESPript/), and the phylogenetic tree is a neighbour-joining tree without distance corrections.

### Transcriptome sequencing of *Talaromyces* sp. F08Z-0631 under different culture conditions

Transcriptome sequencing was performed as previously described (Zhao et al. 2022). *Talaromyces* sp. F08Z-0631 was cultured under three different conditions: high-yield, low-yield and non-yield phlegmacin B1. The high-yield culture conditions are as described above. The medium for low yield was rice and soybean medium without trace elements (the yield of phlegmacin B1 was approximately 50 μg/g medium). The liquid fermentation with 40 mL starch–soybean powder medium (1% starch, 2% glucose, 1% hot-rolled soybean powder, 0.1% KH_2_PO_4_, 0.05% MgSO_4_, 0.2% CaCO_3_, pH not adjusted.) in a 250 mL flask, at 26 ℃, 200 rpm for 7 days (phlegmacin B1 was not detected). The experiments were conducted with three replicates and the mycelia were harvested and ground with liquid nitrogen. Total RNA was extracted with TRIzol (Invitrogen, USA) according to the manufacturer’s protocol. The quality and integrity of RNA samples were determined using a Nanodrop and a Qubit RNA assay kit (Invitrogen, USA). Sequencing libraries were generated and sequenced by Novogene Co., Ltd. (China) on an Illumina HiSeq platform.

### Construction of fungal expression plasmids

The single gene fragments *toaA, toaB* and *toaC* were amplified from *Talaromyces* sp. F08Z-0631 genomic DNA. The corresponding primers used for gene cloning are listed in Additional file 1: Table S1. The PCR products were purified and cloned into the EcoRI and KpnI linearized pTAex3 vectors using the ClonExpress^®^II One Step Cloning Kit according to the manufacturer’s protocol to yield corresponding single gene expression plasmids.

For constructing plasmids coexpressing *toaA* and *toaB*, DNA fragments, including the amylase promoter and terminator, were amplified from corresponding pTAex3-based single gene expression plasmids with the primers listed in Additional file 1: Table S1 and then cloned into SpeI- and PstI-digested pAdeA using the ClonExpress^®^ MultiS One Step Cloning Kit.

The constructed plasmids are listed in Additional file 1: Table S2, and representative plasmid maps are provided in Additional file 1: Fig. S5.

### Construction and fermentation of *A. oryzae* NSAR1 heterologous expression strains

Similar to that previously described (Zhao et al. 2022), transformation of *A. oryzae* NSAR1 was performed by a protoplast–polyethylene glycol method. The spore suspension of *A. oryzae* NSAR1 was seeded in 10 mL DPY medium (2% dextrin, 1% polypeptone, 0.5% yeast extract, 0.05% MgSO_4_·7H_2_O, 0.5% KH_2_PO_4_, without pH adjustment), cultured for 2 days at 30 °C and 150 rpm, and then transferred into 100 mL DPY medium for another day. Mycelia were collected by filtration and digested with 1% Yatalase (Takara, Japan) in 0.6 M (NH_4_)_2_SO_4_, 50 mM maleic acid, pH 5.5, at 30 °C for 3 h. The residues were removed by filtration, and the protoplasts were collected by centrifugation at 1500 rpm for 10 min. Then, the protoplasts were washed with Solution 2 (1.2 M sorbitol, 50 mM CaCl_2_·2H_2_O, 35 mM NaCl, 10 mM Tris–HCl, pH 7.5) and resuspended to approximately 1 × 10^7^ cells mL^−1^ in Solution 2. A total of 10 μL plasmids (5–10 μg) were added into a 200 μL protoplast suspension and incubated on ice for 30 min. Subsequently, 250 μL, 250 μL and 850 μL Solution 3 (60% PEG4000, 50 mM CaCl2·2H2O, 10 mM Tris–HCl, pH 7.5) was added and mixed successively. After incubation at room temperature for 20 min, 10 mL of Solution 2 was added, and the mixture was centrifuged at 1500 rpm for 10 min. The precipitates were suspended in 200 μL of Solution 2 and spread on M medium (0.2% NH_4_Cl, 0.1% (NH_4_)_2_SO_4_, 0.05% KCl, 0.05% NaCl, 0.1% KH_2_PO_4_, 0.05% MgSO_4_·7H_2_O, 0.002% FeSO_4_·7H_2_O, 2% glucose, 1.5% agar) with 1.2 M sorbitol and 0.15% methionine, 0.1%, arginine, which was selected for transformants harbouring the pAdeA-derived plasmid, and only 0.15% methionine at pH 5.5 for harbouring the pAdeA-derived and pTAex3-derived plasmids. Then, the plates were covered with the same upper medium containing 0.8% agar. The plates were incubated at 30 °C for 2–4 days, and the transformants were transferred to the corresponding selection medium without sorbitol for rejuvenation. All of the *A. oryzae* NSAR1 strains constructed in this work are listed in Additional file 1: Table S3.

The *A. oryzae* transformants were cultured in 10 mL DPY medium for 2 days, and then the seed broth was transferred into 100 mL Czapek–Dox (CD) medium (0.3% NaNO_3_, 0.2% KCl, 0.05% MgSO_4_·7H_2_O, 0.1% KH_2_PO_4_, 0.002% FeSO_4_·7H_2_O, 1% polypeptone, 2% starch, pH 5.5) in a 500 mL flask at 30 °C for 5 days and 150 rpm to induce the expression of heterologous genes under the *P*_*amyB*_ promoter. To optimize fermentation conditions, 2% soybean meal and/or 2% peptone were added to CD medium, and then the pH was adjusted to 5.5. The fermentation broth was extracted with ethyl acetate. The extract was concentrated under reduced pressure and resuspended in acetonitrile for the analysis and isolation of metabolites.

Time-course analyses of compound **4** produced by Ao_*toaA/toaB-toaC*_. Spores of Ao_*toaA/toaB-toaC*_ (~ 1 × 10^7^) growing on PDA plates were inoculated into 10 mL DPY medium and cultured at 30 °C in shake flasks with rotary shaking at 150 rpm for 2 days. Then, the seed broth was transferred into 100 mL CD medium in a 500 mL flask at 30 °C for 5 days and 150 rpm. An 800 μL sample of each strain was individually collected each day. The broth was extracted with 800 μL ethyl acetate. The ethyl acetate extraction was dried under a nitrogen flow and redissolved in acetonitrile for the analysis and isolation of metabolites.

### Analysis of metabolites and compound structural characterization

The ethyl acetate extract of fermentation broth and in vitro assays were used for HPLC analysis on an Agilent column (Zobrax SB-C18, 3.5 μm, 2.1 × 50 mm, Agilent, USA). The metabolites were eluted at a flow rate of 0.2 mL/min over a 35 min gradient as follows: *T* = 0 min, 40% B; *T* = 5 min, 40% B; *T* = 20 min, 100% B; *T* = 24 min, 100% B; *T* = 25 min, 40% B; *T* = 35 min, 40% B (solvent A, H2O + 0.1% HCOOH; solvent B, CH3CN + 0.1% HCOOH) with monitoring at 390 nm. For HPLC–ESI–MS analysis, the conditions were the same.

Ethyl acetate extraction was used to isolate compounds from the fermentation of *A. oryzae* transformant strains. Compounds **3** and **4** were isolated from the fermentation of Ao_*toaA/toaB*_ and Ao_*toaA/toaB-toaC*_, respectively. The crude extracts were dried in vacuo and then subjected to C18 column (12 nm S-50 μm) chromatography using a methanol and H_2_O gradient system (from 20% to 100%). Fractions containing the target compound were combined for further purification using a semiprep HPLC with a C18 column (Agilent ZORBAX SB-C18, 5 μm, 9.4 × 250 mm). For structural characterization, HRESIMS, NMR, and optical rotation of compounds **3** and **4** were recorded. The structures of isolated compounds were determined by comparison with published data (Additional file 1: Text, Figs. S6, S7, and Tables S4, S5). The titres of compounds **3** and **4** were evaluated based on the HPLC peak area relative to pure compounds separated.

### Exploration of the conversion of tetrahydroanthracene to anthraquinone compounds by alkali treatment

Compounds **3** and **4** (1 mg/mL) of dissolved in acetonitrile were divided into five 1.5 mL centrifuge tubes, each 100 µL, and dried with a termovap sample concentrator. Then, Tube 1: add 200 µL ddH2O; Tube 2: add 200 µL 0.1% NaOH; Tube 3, add 200 µL 1% NaOH; Tube 4, add 200 µL 5% NaOH; Tube 5, add 200 µL 5% NaOH. The centrifuge tubes were placed at room temperature for reaction for 3 h, and the pH value of tubes 2–4 was adjusted to 4–5 with 1% HCl. Subsequently, all 5 tubes of samples were fixed to 500 µL with ddH_2_O, extracted with 600 µL ethyl acetate and centrifuged at 12,000 rpm for 5 min. The supernatant was dried with a termovap sample concentrator and then dissolved in 500 µL acetonitrile for HPLC or HPLC–HRMS analysis.

### Producing anthraquinone compounds by alkali treatment of the fermentation broth engineered *A. oryzae* NSAR1 strains

One hundred millilitres of the fermentation broth of Ao_*toaA/toaB-toaC*_ incubated in CD medium with 2% soybean meal at 30 °C, and 150 rpm for 2 days, then 5 g solid NaOH (final concentration: 50 g/L) was directly added to the broth, shaken and mixed, and left at room temperature for 24 h. Most of the mycelia were lysed, and the fermentation broth became dark reddish brown. The remaining mycelia were removed by filtration with filter paper, diluted with 1.5 times the volume of ddH_2_O, and then extracted twice with an equal volume of ethyl acetate. The extract was vacuumed, and the crude physcion was obtained. The titres of physcion were evaluated based on the HPLC peak area relative to their authentic standards. The HPLC purity of physion was calculated from the peak area of 390 nm wavelength. Three independent replicates were performed.

### Statistical analyses

The experimental data were analyzed by GraphPad Prism 5, and the significant differences were analyzed using two-way ANOVA.

## Results and discussion

### Construction of a high-yield strain of 3R-torosachrysone (4)

Recently, in the exploration of the biosynthesis of the dihydroanthracenone natural products phlegmacins, we demonstrated that they were formed by the specific unsymmetrical 7,10ʹ coupling of two torosachrysone (**4**) monomers catalysed by the laccase PhlC and Fas protein PhlB colocated in the mitochondrial membrane in the ascomycete *Talaromyces* sp. F08Z-0631. We successfully reconstructed the biosynthetic pathway of the monomer torosachrysone in *A. oryzae* through the heterologous expression of three genes: the ACAS gene *phlD* and ACTE gene *phlA* in the gene cluster and the heterologous methyltransferase gene *aurJ* that is responsible for C6 *O*-methylation of anthracene nor-rubrofusarin to form rubrofusarin in the biosynthesis of aurofusarin in *Fusarium graminearum *(Zhao et al. 2022)*.* Using ACAS (accession no. XP_001217072.1) and ACTE (accession no. XP_001217071) from *A. terreus* as probes, in addition to the phlegmacins gene cluster, another gene cluster, composed only of an NR-iPKS (*toaA*) and a MβTE (*toaB*), was found at the same time in the genome of the fungus *Talaromyces* sp. F08Z-0631. The similarity/identity of ToaA to PhlD is 44%/60% and that of ToaB to PhlA is 47%/62% (Additional file 1: Figs. S1–S3). This *toa* gene cluster is a silenced gene cluster and was not expressed under the whatever conditions of high, low or no yield of phlegmacins (Additional file 1: Fig. S4).

**Fig. 2:**
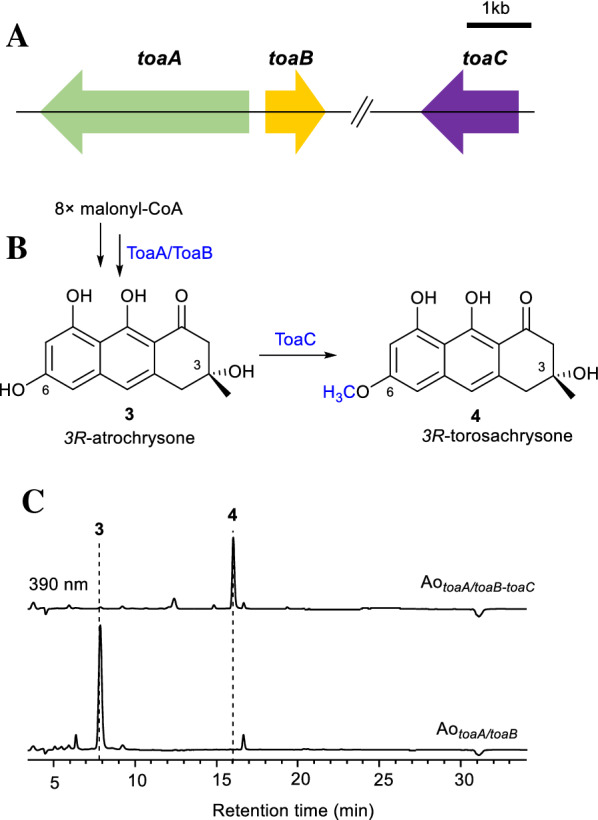
Heterologous biosynthesis of tetrahydroanthracene *3R*-atrochrysone **3** and *3R*-torosachrysone **4** in *Aspergillus oryzae.* **A** Silent *toa* gene cluster and the separated methyltransferase ToaC. **B** Biosynthetic pathway of *3R*-torosachrysone **4**. **C** Metabolite analysis of culture extracts of *A. oryzae* heterologous expression strains by HPLC (λ = 390 nm)

NR-iPKS lacks a thioesterase or Claisen-like cyclase (TE/CLC) domain to cyclize and release the products but has a stand-alone MβTE, representing a clade of fungal PKSs that are linked to the biosynthesis of various polycyclic aromatic polyketides, such as SpoP/SpoB (Thiele et al. 2020b) and ShwP/ShwB (Thiele et al. 2020a) forming naphthalene 6-hydroxymusizin; MdpG/MdpF (Chiang et al. 2010; Schätzle et al. 2012), RugA/RugB (Han et al. 2021), ClaG/ClaF (Griffiths et al. 2016), ACAS/ACTE (Awakawa et al. 2009), PhlD/PhlA (Zhao et al. 2022) and AgnPKS/AgnL7 (Szwalbe et al. 2019) forming tetrahydroanthracene atrochrysone; AdaA/AdaB (Li et al. 2011) in the biosynthetic gene cluster of linear tetracyclic TAN-1612; and VrtA/VrtG (Chooi et al. 2010) forming the tetracyclic skeleton in the biosynthesis of viridicatumtoxin (Additional file 1: Fig. S1A). However, these enzymes have high similarity, and it is difficult to predict their products by sequence alignment or phylogenetic tree analysis (Additional file 1: Figs. S1–S3). To understand the function of the *toa* gene cluster, the *toaA* and *toaB* genes were simultaneously cloned with the amylase promoter/terminator PamyB/TamyB in the pAdeA vector harbouring the *adeA* selective marker. The resulting plasmid pAdeA-*toaA/toaB* (Additional file 1: Fig. S5) was introduced via glycol polyethylene glycol (PEG)-mediated transformation into the adenine auxotroph *A. oryzae* NSAR1 for heterologous expression. After starch-induced expression for 4 days, the Ao_*toaA/toaB*_ extract was analysed by high-performance liquid chromatography (HPLC), and an additional compound (**3)** was detected and isolated (Fig. 2C). Unexpectedly, according to high-resolution electrospray ionization mass spectrometry (HRESIMS), nuclear magnetic resonance (NMR), and optical rotation measurements (Additional file 1: Text, Fig. S6, and Table S4), compound **3** was established as *3R*-atrochrysone, completely consistent with the compound produced by expressing *phlD* and *phlA* from the biosynthetic gene cluster of phlegmacins. Moreover, the yield of *3R*-atrochrysone was much higher (1.93-fold) in Ao_*toaA/toaB*_, than in Ao_*phlD/phlA,*_ and reached 2.49 g/L by shake flask fermentation.

In the genome of *Talaromyces* sp. F08Z-0631, we found 8 homologues of the methyltransferase AurJ. Among them, the one with the highest identity/similarity with AurJ was named ToaC. The *toaC* gene was cloned into the pTAex3 vector with the amylase promoter/terminator PamyB/TamyB. The plasmid pTAex3-*toaC* was transformed into Ao_*toaA/toaB,*_ and the resulting strain Ao_*toaA/toaB-toaC*_ produced compound **4** (Additional file 1: Fig. S2C), which was isolated and identified as *3R*-torosachrysone by HRESIMS, NMR, and optical rotation measurements (Additional file 1: Text, Fig. S7, and Table S5). ToaC was thus identified as a specific atrochrysone C6–OH methyltransferase. The shake flask fermentation yield of torosachrysone produced by Ao_*toaA/toaB-toaC*_ reached 0.84 g/L, which is 2.46 times that obtained from Ao_*phlD/phlA-aurJ*_.

### Conversion of 3R-torosachrysone (4) into physcion (1)

**Fig. 3:**
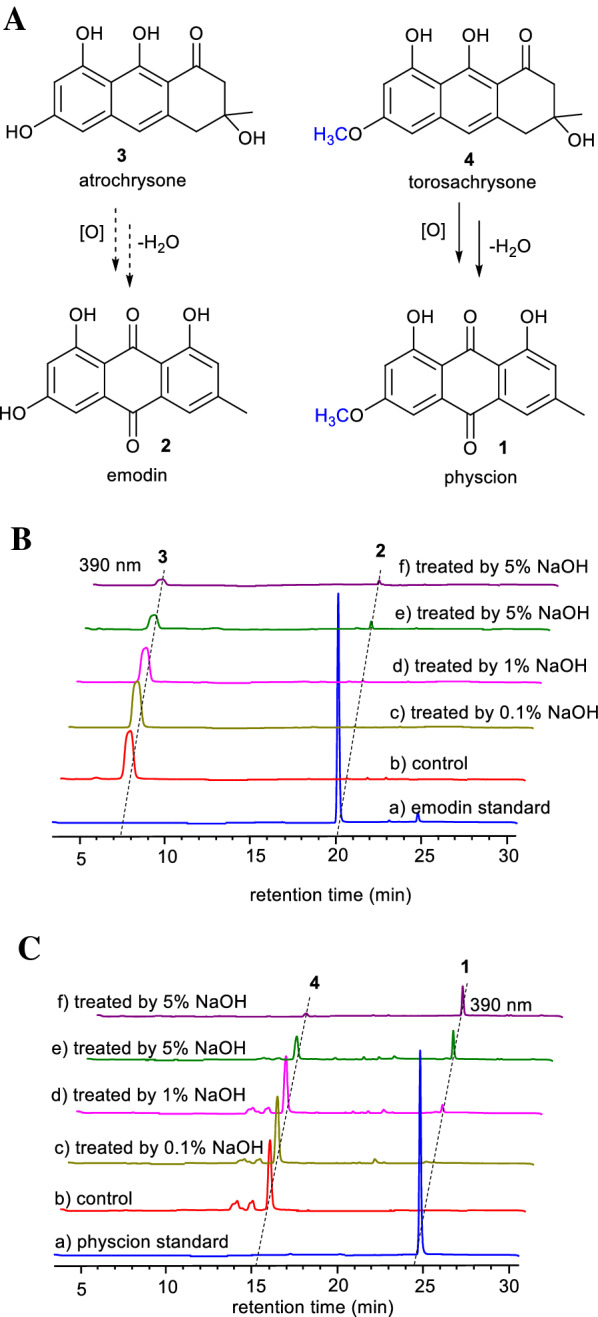
Tetrahydroanthracenes **3** and **4** were converted to anthraquinones **2** and **1** by alkali treatment at different concentrations. **A** Scheme of conversion. **B** HPLC analysis of the alkali treatment products of **3**: **a** commercial emodin standard; **b**–**e** **3** treated with 0%, 0.1%, 1%, and 5% NaOH aqueous solution, with the pH value adjusted to 4–5 before extraction; **f** **3** treated with 5% NaOH aqueous solution without pH adjustment. **C** HPLC analysis of the alkali treatment products of **4**: **a** commercial physcion standard; **b**–**e** **4** treated with 0%, 0.1%, 1%, and 5% NaOH aqueous solution, with the pH value adjusted to 4–5 before extraction; **f** **4** treated with 5% NaOH aqueous solution without pH adjustment

Next, chemical transformation was conducted to convert tetrahydroanthracene products **3** and **4** to the more valuable anthraquinone compounds **2** and **1**. When **3** or **4** was directly dissolved in acetonitrile overnight, trace amounts of **2** or **1,** respectively, could be detected (Additional file 1: Fig. S8). This indicated that tetrahydroanthracene can spontaneously oxidize and dehydrate to form anthraquinone under mild neutral conditions, but the efficiency is very low. Thus, we tried to accelerate the conversion process by alkali treatment at different concentrations. A solution containing 1 mg/mL compound **3** or **4** dissolved in acetonitrile was divided into 100 µL aliquots, and dried with a thermovap sample concentrator, and then 200 µL of 0%, 0.1%, 1%, or 5% NaOH solution was added. Although these two compounds are almost insoluble in pure water, both are highly soluble in alkali solution. Considering that anthracenes are usually not easily extracted by organic solvents under alkaline conditions, we used 1% hydrochloric acid to adjust the pH to 4–5 after allowing the solution to stand at room temperature for 3 h. Then, the reaction product was extracted with ethyl acetate, dried and dissolved in acetonitrile, followed by HPLC analysis. The results showed that with increasing alkali concentration, the amount of tetrahydroanthracene substrate **4** decreased, and the yield of physcion **1** increased significantly. Under the same conditions, although **3** was also significantly reduced, surprisingly, only trace amounts of emodin **2** were produced (Fig. 3A). We speculate that compound **3,** compared with compound **4**, may form more byproducts in the alkali solution. Slightly surprisingly, when extracted by ethyl acetate directly under extremely alkaline conditions without pH adjustment, physcion **1** could be extracted effectively, while other impurities and byproducts were almost all in the aqueous layer (Fig. 3Cf).

### Production of physcion from the fermentation broth of engineered strain Ao_toaA/toaB-toaC_

**Fig. 4:**
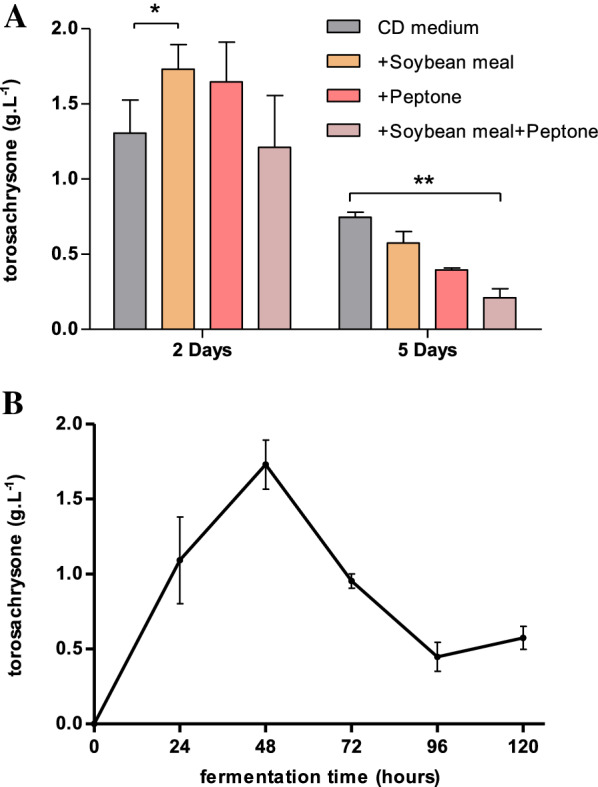
Optimizing the fermentation conditions of the engineered torosachrysone (**4**)-producing strain Ao_*toaA/toaB–toaC*_. **A** Effects of different nitrogen sources in fermentation CD medium on compound **4** production. *p ≤ 0.05, **p ≤ 0.01. **B** Fermentation time curve of compound **4** production with CD medium + 2% soybean meal. Error bars represent the standard deviations of three identical replicates

To obtain higher yield of physcion, the fermentation conditions of the engineered compound **4**-producing strain Ao_*toaA/toaB-toaC*_ were optimized. We attempted to supplement the relatively undernourished CD-starch medium with nitrogen sources soybean meal and/or peptone and discovered that while the yield of compound **4** did not increase but rather decreased after a long fermentation period (5 days), it could significantly increase the yield during a short period of fermentation (2 days), especially when adding soybean meal, which could increase the yield to 1.73 g/L (Fig. 4A). Then, we evaluated the fermentation time curve under the condition of adding soybean meal and found that the output of compound **4** peaked in 2 days and then abruptly decreased, likely as a result of fungal degradation (Fig. 4B).

We directly treated the fermentation broth of the torosachrysone **4** high-yield engineered strain Ao_*toaA/toaB-toaC*_ according to the above results and optimized conditions. Solid NaOH (final concentration 50 g/L) was added to the fermentation broth of Ao_*toaA/toaB-toaC*_ that had been incubated in CD medium with 2% soybean meal at 30 °C for 2 days. The majority of the mycelia were lysed and the fermentation broth turned dark reddish brown after 24 h at room temperature. The residual mycelia were removed and extracted with ethyl acetate. After vacuum drying, 526 ± 2.5 mg of physcion **1** with 94.8% HPLC purity was directly obtained from each litre of shake flask fermentation broth.

In summary, we constructed an *A. oryzae* strain with a high yield of *3R*-torosachrysone (**4**), the tetrahydroanthracene precursor of physcion, by heterologous expression of related genes mined from the phlegmacins-producing ascomycete *Talaromyces* sp. F08Z-0631. Studying and optimizing the conditions for converting **4** into physcion led to the development of a concise strategy for efficient manufacturing the phytogenic biopesticide physcion by microbial fermentation and straightforward chemical post-treatment. This method has a great potential for industrial scale up and serves as an example for the microbial production of other anthraquinone compounds originating from plants.

### Supplementary Information

**Additional file 1: Text** Physicochemical properties and structural elucidation of atrochrysone (**3**) and torosachrysone (**4**). **Fig. S1.** HPLC analysis of the metabolic profile of *Talaromyces *sp*.* F08Z-0631 at 390 nm. **Fig. S2.** Sequence alignment of the characterized fungal NR-iPKS with a stand-alone MβL-TE.** Fig. S3.** Sequence alignment of characterized MβL-TE.** Fig. S4.** Transcriptome analysis of *toaA–toaC* and genes in the BGC of phlegmacins from* Talaromyces* sp. F08Z-0631 under different fermentation conditions.** Fig. S5.** The plasmid maps of pAdeA-*toaA/toaB *and pTAex3-*toaC. Fig. S6.* ^1^H NMR spectrum of atrochrysone **3**.** Fig. S7.** ^1^H NMR spectrum of torosachrysone **4**. **Fig. S8.** HPLC analysis of the metabolites of compounds **3** and **4** directly dissolved in acetonitrile overnight. **Table S1.** Primers used in this study. **Table S2.** Plasmids used in the study.** Table S3.** Strains used in the study.** Table S4.** ^1^H NMR signals of isolated Compound **3** compared to previously identified atrochrysone. **Table S5.** ^1^H NMR signals of isolated Compound **4** compared to previously identified torosachrysone.

## Data Availability

The sequences of the *toaA, toaB* and *toaC* genes were deposited in GenBank with accession numbers ON979688, ON979689 and ON979690. Experimental materials and data sets for the current study are available from the corresponding author on reasonable request.
