# Supplementary material for: Production of the antifungal biopesticide physcion through the combination of microbial fermentation and chemical post-treatment
Source: Bioresour Bioprocess. 2023 Jan 9;10(1):2. doi: 10.1186/s40643-023-00625-8 (PMC10991666; doi:10.1186/s40643-023-00625-8)
Supplement: Supplementary file 1 — Additional file 1: Text Physicochemical properties and structural elucidation of atrochrysone (3) and torosachrysone (4). Fig. S1. HPLC analysis of the metabolic profile of Talaromyces sp. F08Z-0631 at 390 nm. Fig. S2. Sequence alignment of the characterized fungal NR-iPKS with a stand-alone MβL-TE. Fig. S3. Sequence alignment of characterized MβL-TE. Fig. S4. Transcriptome analysis of toaA–toaC and genes in the BGC of phlegmacins from Talaromyces sp. F08Z-0631 under different fermentation conditions. Fig. S5. The plasmid maps of pAdeA-toaA/toaB and pTAex3-toaC. Fig. S6. 1H NMR spectrum of atrochrysone 3. Fig. S7. 1H NMR spectrum of torosachrysone 4. Fig. S8. HPLC analysis of the metabolites of compounds 3 and 4 directly dissolved in acetonitrile overnight. Table S1. Primers used in this study. Table S2. Plasmids used in the study. Table S3. Strains used in the study. Table S4. 1H NMR signals of isolated Compound 3 compared to previously identified atrochrysone. Table S5. 1H NMR signals of isolated Compound 4 compared to previously identified torosachrysone. [file 40643_2023_625_MOESM1_ESM.pdf]

Additional file

Production of the Antifungal Biopesticide Physcion through the Combination of Microbial Fermentation and Chemical Posttreatment

Zheng Zhuang[1], Xueqing Zhong[1], Qinghua Li[1], Tian Liu[4], Qing Yang[2], Guo-Qiang Lin[1], Qing-Li He[1*], Qunfei Zhao[1*] and Wen Liu[3]

[1] The Research Center of Chiral Drugs, Innovation Research Institute of Traditional Chinese Medicine, Shanghai University of Traditional Chinese Medicine, 1200 Cailun Road, Shanghai 201203, China.

[2] State Key Laboratory for Biology of Plant Diseases and Insect Pests, Institute of Plant Protection, Chinese Academy of Agricultural Sciences, No. 2 West Yuanmingyuan Road, Beijing 100193, China

[3] State Key Laboratory of Bioorganic and Natural Products Chemistry, Shanghai Institute of Organic Chemistry, Chinese Academy of Sciences, 345 Lingling Road, Shanghai 200032, China

[4] School of Bioengineering, Dalian University of Technology, No. 2, Linggong Road, Dalian 116024, China

Correspondence:

qinglihe@shutcm.edu.cn (Q.H.),
qunfeizhao@shutcm.edu.cn (Q.Z.),

Table of Contents

1. Additional file Text

1.1 Physico-Chemical Properties and Structural Elucidation of Atrochrysone (**3**)

1.2 Physico-Chemical Properties and Structural Elucidation of Torosachrysone (**4**)

2. Additional file Figures

Figure S1. HPLC analysis of the metabolic profile of *Talaromyces sp.* F08Z-0631at 390 nm

Figure S2. Sequence alignment of the characterized fungal NR-iPKS with a stand-alone MβL-TE.

Figure S3. Sequence alignment of characterized MβL-TE.

Figure S4. Transcriptome analysis of *toaA-toaC* and genes in the BGC of phlegmacins from *Talaromyces* sp. F08Z-0631 under different fermentation conditions.

Figure S5. The plasmid maps of pAdeA-*toaA/toaB* and pTAex3-*toaC.*

Figure S6. ^{1}H NMR spectrum of atrochrysone **3.**

Figure S7. ^{1}H NMR spectrum of torosachrysone **4.**

Figure S8. HPLC analysis of the metabolites of compound **3** and **4** directly dissolved in acetonitrile overnight.

3. Additional file Tables

Table S1. Primers used in this study

Table S2. Plasmids used in the study

Table S3. Strains used in the study

Table S4. ^{1}H NMR signals of isolated Compound **3** compared to previously identified atrochrysone.

Table S5. ^{1}H NMR signals of isolated Compound **4** compared to previously identified torosachrysone.

4. Additional file References

1. Additional file Text

1.1 Physico-Chemical Properties and Structural Elucidation of Atrochrysone (3).

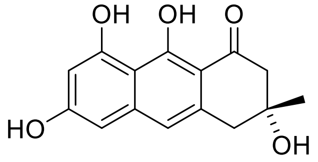

3R-atrochrysone (**3**) was purified as green-yellow solid: UV $_{(MeOH)}$ λ_{max} 271 nm; $[\alpha]_D^{30.1}$ = -10.05 [MeOH; c 0.4]. ^{1}H NMR (600 MHz, Acetone-d_6) see **Supplementary Table 4** and **Figure S6**; ESI-HR-MS Calcd. for $C_{15}H_{13}O_5^-$ 273.0768 [M-H]$^-$, found 273.0763. The molecular formula of Compound **3** was established to be $C_{15}H_{14}O_5$. By analyzing its ESI-HR-MS, ^{1}H NMR spectra and optical rotation, his compound is consistent with *3R*-atrochrysone, which we previously characterized in the study of the heterologous biosynthesis of phlegmcins.[1]

2.2 Physico-Chemical Properties and Structural Elucidation of Torosachrysone (4).

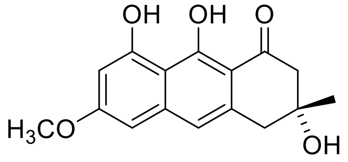

3R-torosachrysone (**4**) was purified as citrine solid: UV $_{(MeOH)}$ λ_{max} 271 nm; $[\alpha]_D^{30.1}$ = -4.9 [MeOH; c 0.2]; ^{1}H NMR (600MHz, $CDCl_3$-*d*) see **Supplementary Table 5** and **Figure S7**; ESI-HR-MS Calcd. for $C_{16}H_{15}O_5^-$ 287.0925 [M-H]$^-$, found 287.091. The molecular formula of Compound **4** was established to be $C_{15}H_{16}O_5$. By analyzing its ESI-HR-MS, ^{1}H NMR spectra and optical rotation, his compound is consistent with *3R*-torosachrysone, which we previously characterized in the study of the heterologous biosynthesis of phlegmcins.[1]

2. Additional file Figures

A.

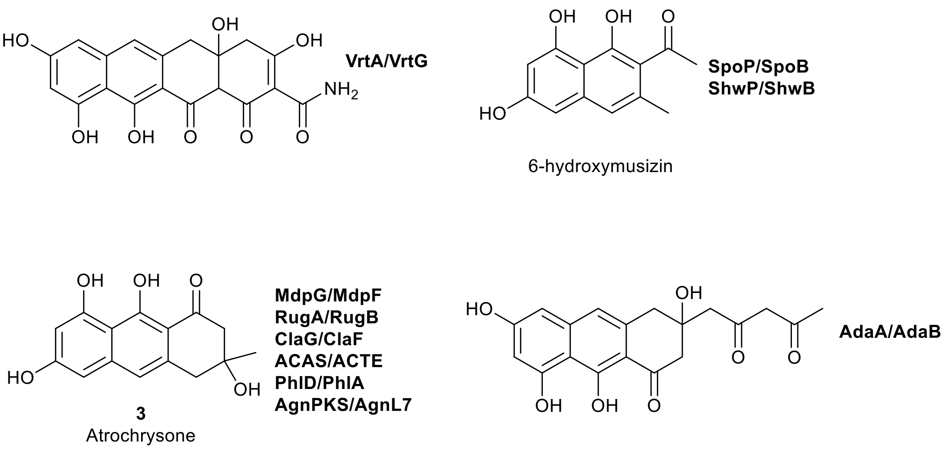

B.

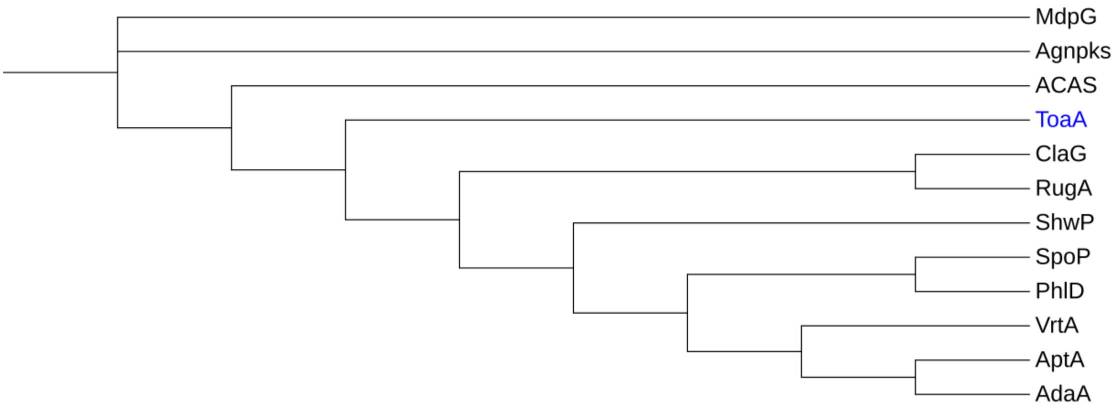

C.

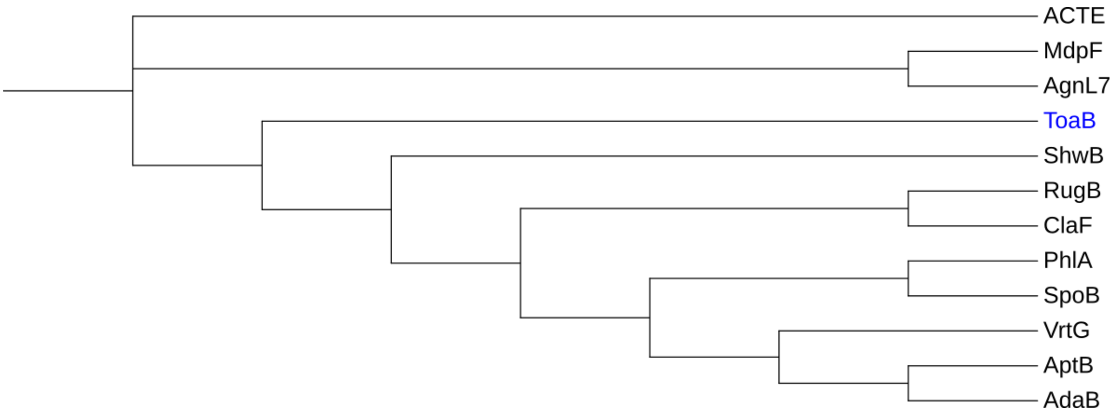

Figure S1. The characterized fungal NR-iPKS with a stand-alone MβL-TE in the literature: VrtA/VrtG[2], SpoP/SpoB[3], ShwP/ShwB[4], AdaA/AdaB[5], MdpG/MdpF[6-7], RugA/RugB[8], ClaG/ClaF[9], ACAS/ACTE[10], PhlD/PhlA[1], AgnPKS/AgnL7[11]. **A.** Products formed by fungal NR-iPKS with a stand-alone MβL-TE; **B.** Phylogenetic tree of NR-iPKS; **C.** Phylogenetic tree of MβL-TE.

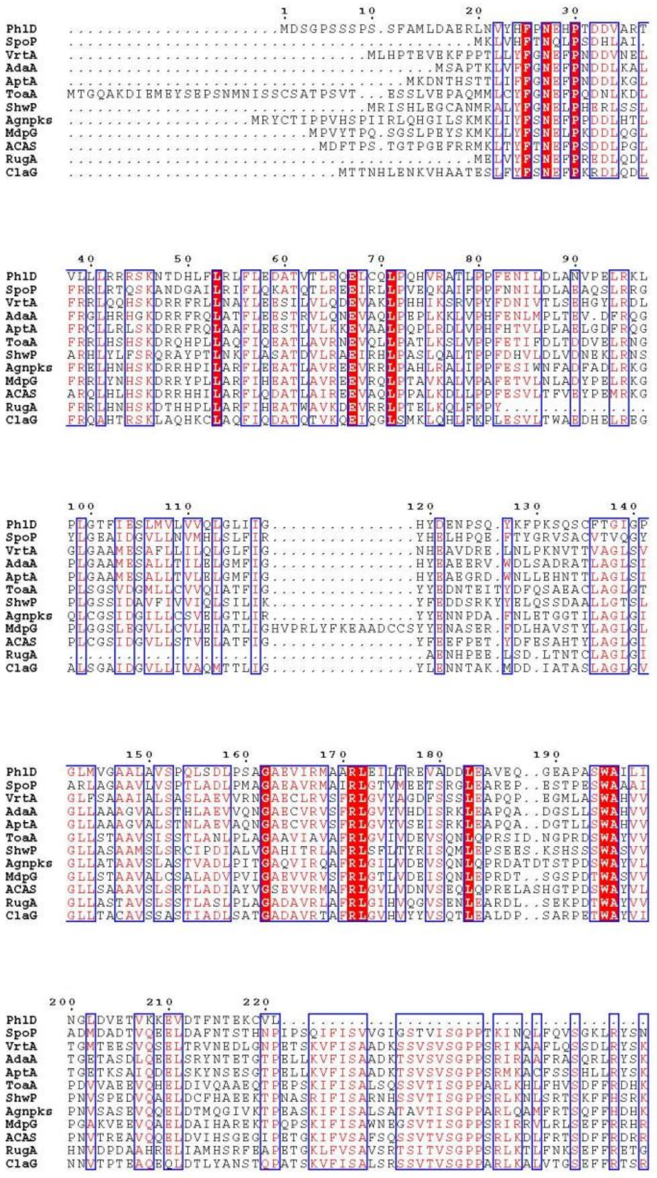

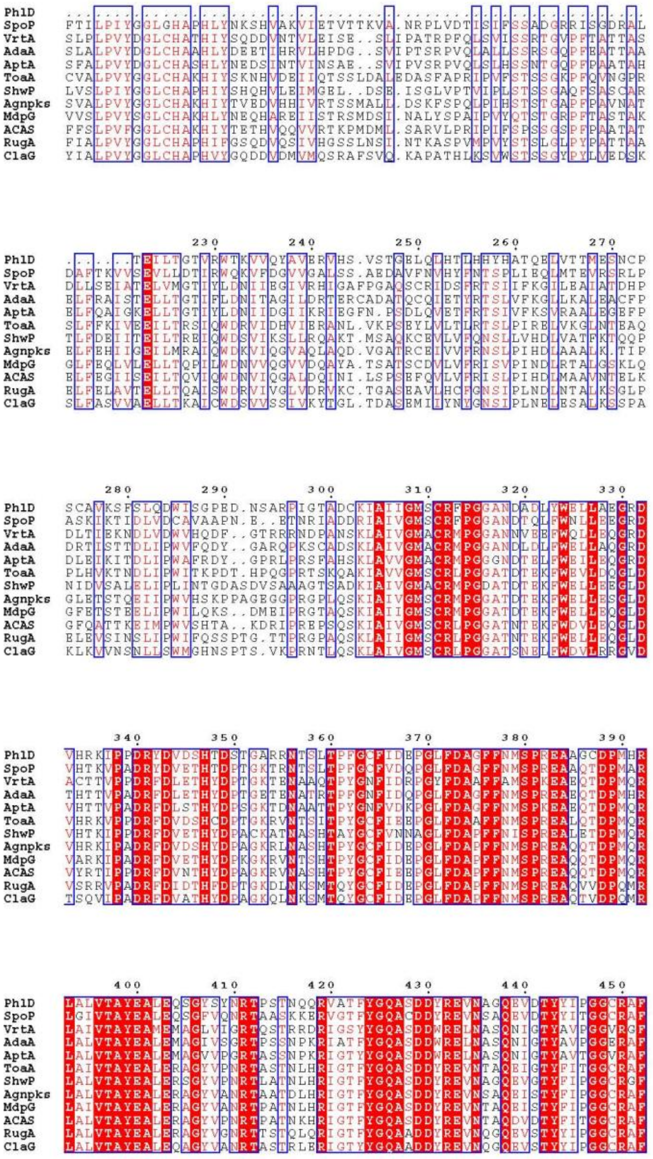

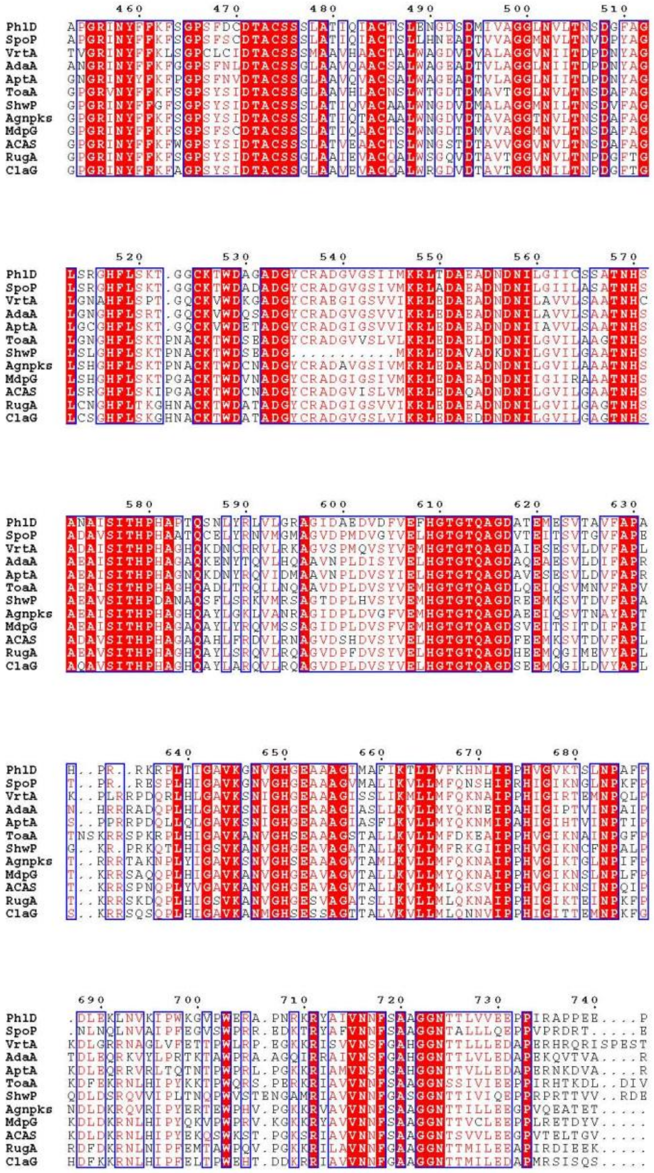
460 470 480 490 500 510
520 530 540 550 560 570
580 590 600 610 620 630
640 650 660 670 680
690 700 710 720 730 740
Ph1D
SpoP
VrtA
AdaA
AptA
ToaA
ShwP
Agnpks
MdpG
ACAS
RugA
ClaG

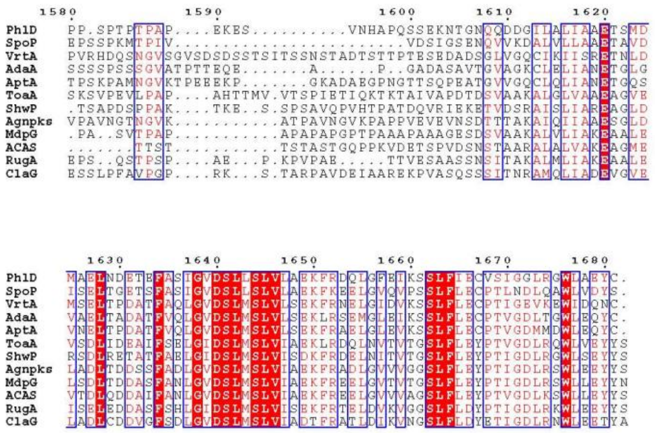

Figure S2. Sequence alignment of the characterized fungal NR-iPKS with a stand-alone MβL-TE: VrtA[2], SpoP[3], ShwP[4], AptA[12], AdaA[5], MdpG[6-7], RugA[8], ClaG[9], ACAS[10], PhlD[1], AgnPKS[11].

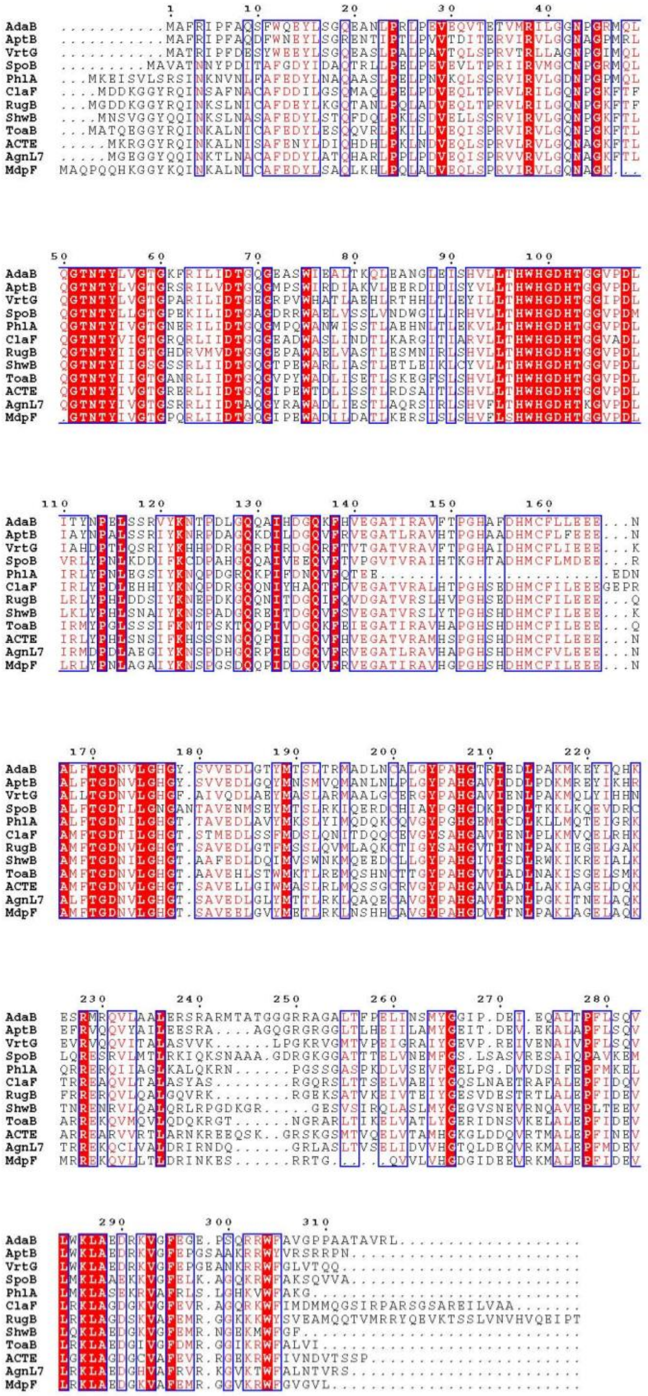

Figure S3. Sequence alignment of characterized MβL-TE: VrtG[2], SpoB[3], ShwB[4], AptB[12], AdaB[5], MdpF[6-7], RugB[8], ClaF[9], ACTE[10], PhlA[1], AgnL7[11].

.

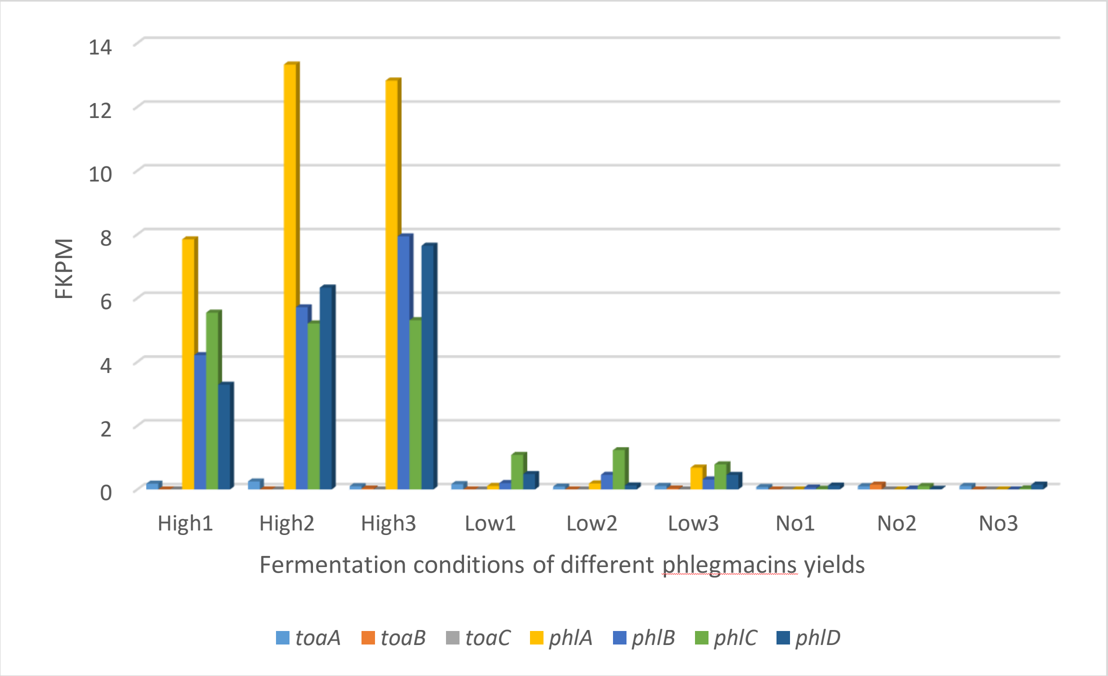

Figure S4. Transcriptome analysis of *toaA*-*toaC* and genes in the BGC of phlegmacins from *Talaromyces* sp. F08Z-0631 under different fermentation conditions. FPKM: fragments per kilobase per million. High1, High2 and High3: three transcripts under high-yield fermentation conditions. Low1, Low2 and Low3: three transcripts under low-yield fermentation conditions. No1, No2 and No3: three transcripts under the fermentation conditions that phlegmacins was not produced.

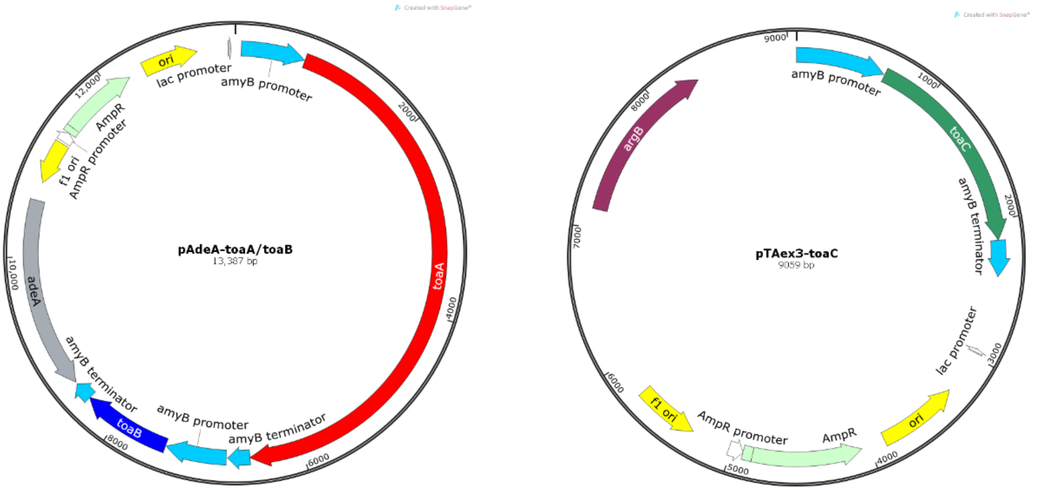

Figure S5. The plasmid maps of pAdeA-*toaA/toaB* and pTAex3-*toaC*.

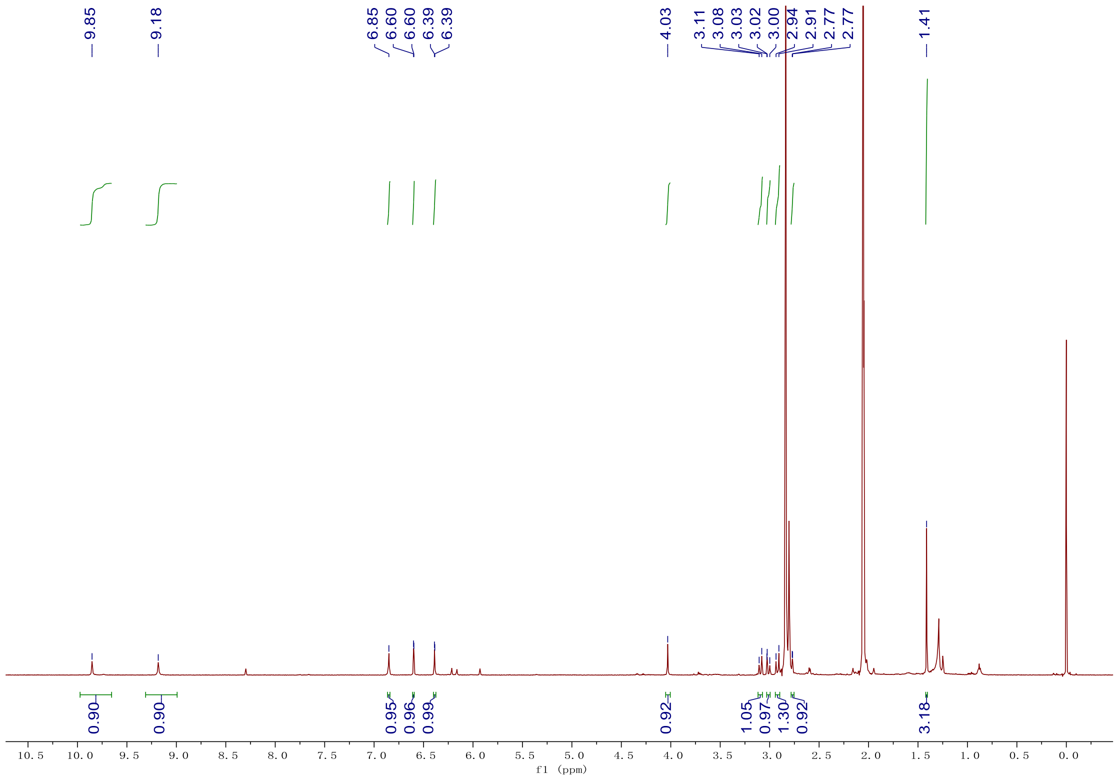

Figure S6. ^{1}H NMR spectrum of atrochrysone **3.**

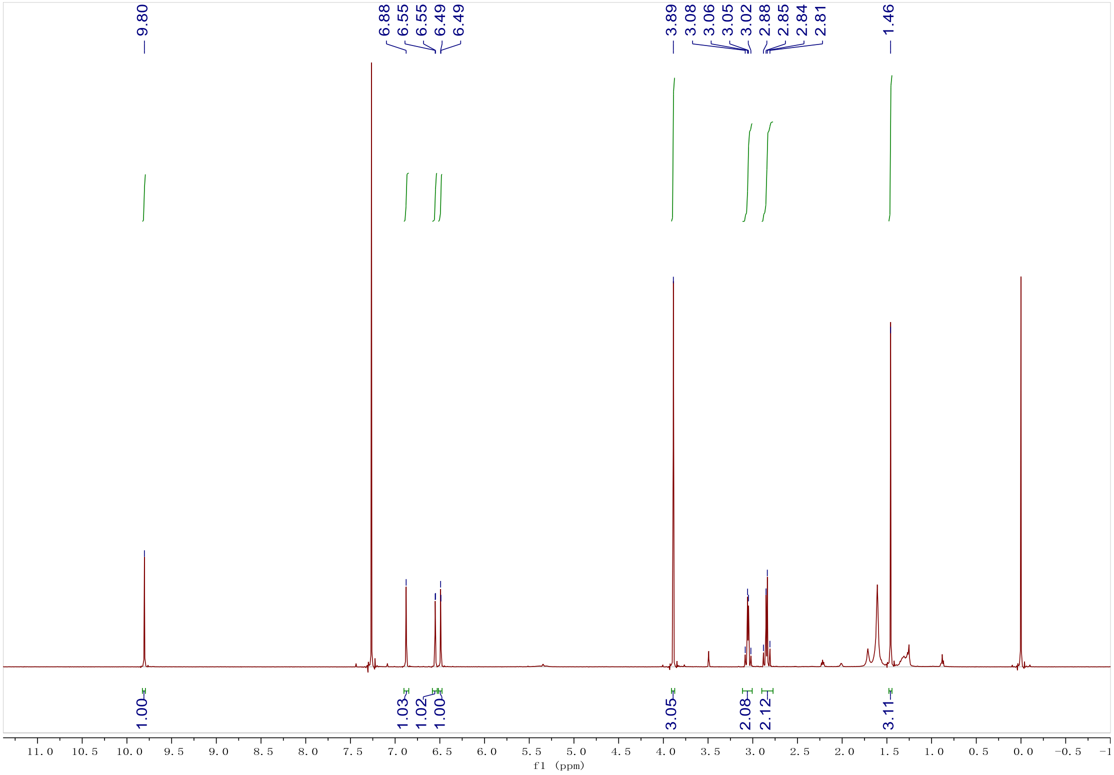

Figure S7. ^{1}H NMR spectrum of torosachrysone **4.**

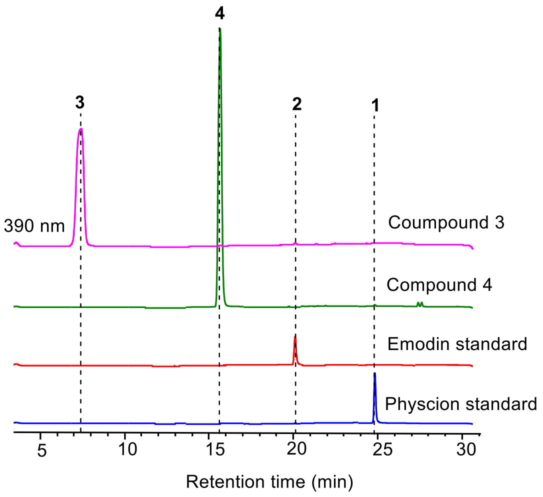

Figure S8. HPLC analysis of the metabolites of compound **3** and **4** directly dissolved in acetonitrile overnight.

3. Additional file Tables

Table S1. Primers used in this Study.

**Primer**	**Sequence (5' to 3')**	**Usage**
pTAex3-*toaA*-F	AGCAAGCTCCGAATTCGAAACAA TGACTGGACAGGCCAAGGATATC	Cloning of *toaA* from *Talaromyces* sp. F08Z-0631genome
pTAex3-*toaA*-R	ACTACAGATCCCCGGTACCCTAGC TGTAATACTCCACCA	
pTAex3-*toaB*-F	AGCAAGCTCCGAATTCGAAACAA TGGCAACCCAAGAAGGGGG	Cloning of *toaB* from *Talaromyces* sp. F08Z-0631genome
pTAex3-*toaB*-R	ACTACAGATCCCCGGTACCTCAAA TTACAAGGGCGAACCAC	
pTAex3-*toaC*-F	AGCAAGCTCCGAATTCACCATGG CTGGAACTTCTAATCAAG	Cloning of *toaC* from *Talaromyces* sp. F08Z-0631genome
pTAex3-*toaC*-R	ACTACAGATCCCCGGTCACTCCAA CGACTGCAATC	
Parm-F1	CGTCGCGAGAGCGTTCCACTGCAT CATCAGTCTAG	Construction of recombinant pAdeA plasmids containing two exogenous genes
Tamy-R1	AACGCTCTCGCGACG AAGTACCATACAGTACCGCG	
pAdeA-Pamy-F	GGAAAGCTTGCATGCCTGCAGCG ACTCCAATCTTCAAGAGC	Construction of recombinant pAdeA plasmids containing two exogenous genes from pTAex3
pAdeA-Tamy-R	CGCAGAATCCATATGACTAGTGTA AGATACATGAGCTTCGG	

Table S2. Plasmids used in the study

**Plasmid**	**Characteristic(s)**	**Sources/ Reference**
pTAex3	Plasmid containing *argB* maker gene cassette for gene expression in *A. oryzae* NSAR1	[13]
pAdeA	Plasmid containing *adeA* maker gene cassette for gene expression in *A. oryzae* NSAR1,	[14]
pTAex3-*toaA*	pTAex3 containing *toaA*, expression is regulated by amyB promoter	This study
pTAex3-*toaB*	pTAex3 containing *toaB*, expression is regulated by amyB promoter	This study
pTAex3-*toaC*	pTAex3 containing *toaC*, expression is regulated by amyB promoter	This study
pAdeA-*toaA*/*toaB*	pAdeA containing *toaA* and *toaB*, expressions are independently regulated by *amyB* promoter	This study

Table S3. Strains used in the study

**Strain/Plasmid**	**Characteristic(s)**	**Sources/ Reference**
Talaromyces sp. F08Z-0631	Phlegmacins-producing strain	[15]
A. oryzae NSAR1	Host for gene expression, a quadruple auxotrophic mutant strain (*niaD*⁻, *sC*⁻, *ΔargB*, *adeA*⁻)	[14]
$Ao_{toaA/toaB}$	*A. oryzae* NSAR1 transformant containing pAdeA-*toaA*/*toaB*	This study
$Ao_{toaA/toaB\text{-}toaC}$	*A. oryzae* NSAR1 transformant containing pAdeA-*toaA/toaB* and pTAex3-*toaA/toaB-toaC*	This study
E. coli DH5α	Host for general cloning	Invitrogen

Table S4. H NMR signals of isolated Compound **3** compared to previously identified atrochrysone[1].

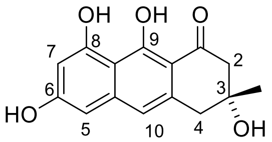

Position	^{1}H NMR (600 MHz, Acetone-d_6)	Previously identified atrochrysone (600 MHz Acetone-d_6)[1]
2	2.77 (d, J = 2.0 Hz);	2.78 (dd, J = 2.0, 17.3Hz);
	2.92 (d, J = 17.3 Hz)	2.92 (d, J = 17.3 Hz)
3-CH_3	1.41, s	1.41, s
3-OH	4.03, s	4.05, s
4	3.01 (d, J = 15.7 Hz)	3.03– 2.99 (m, 1H)
	3.09 (d, J = 15.9 Hz)	3.09 (d, J = 15.8 Hz)
5	6.60 (d, J = 2.1 Hz)	6.60 (d, J = 2.2 Hz)
6-OH	9.18, s	9.20, s
7	6.39 (d, J = 2.1 Hz)	6.39 (d, J = 2.2 Hz)
8-OH	9.85, s	9.85, s
9-OH	/	/
10	6.85, s	6.85, s

Table S5. ^{1}H NMR signals of isolated Compound **4** compared to previously identified torosachrysone [1].

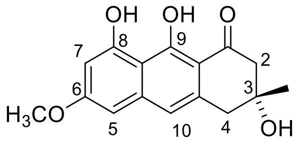

Position	^{1}H NMR (600 MHz, $CDCl_3$-*d*)	Previously identified torosachrysone (600 MHz, $CDCl_3$-*d*)[1]
2	2.90 – 2.77 (m, 2H)	2.90 – 2.80 (m, 2H)
3-CH_3	1.46, s	1.41, s
4	3.11 – 3.01 (m, 2H)	3.10 – 3.01 (m, 2H)
5	6.55 (d, *J* = 2.2 Hz)	6.55 (d, *J* = 2.2 Hz)
6-OCH_3	3.89, s	3.89, s
7	6.49 (d, *J* = 2.2 Hz)	6.49 (d, *J* = 2.2 Hz)
8-OH	9.80, s	9.81, s
9-OH	/	/
10	6.88, s	6.88, s

4. Additional file References

1. *Zhao, Q.; Zhuang, Z.; Liu, T.; Yang, Q.; He, Q. L.; Liu, W.; Lin, G. Q., Unsymmetrically Regioselective Homodimerization Depends on the Subcellular Colocalization of Laccase/Fasciclin Protein in the Biosynthesis of Phlegmacins. ACS Chem. Biol.* ***2022,*** *17, 791-796.*
2. *Chooi, Y. H.; Cacho, R.; Tang, Y., Identification of the viridicatumtoxin and griseofulvin gene clusters from Penicillium aethiopicum. Chem. Biol.* ***2010,*** *17 (5), 483-94.*
3. *Thiele, W.; Obermaier, S.; Müller, M., A Fasciclin Protein Is Essential for Laccase-Mediated Selective Phenol Coupling in Sporandol Biosynthesis. ACS Chem. Biol.* ***2020,*** *15 (4), 844-848.*
4. *Thiele, W.; Froede, R.; Steglich, W.; Müller, M., Enzymatic Formation of Rufoschweinitzin, a Binaphthalene from the Basidiomycete Cortinarius rufoolivaceus. Chembiochem* ***2020,*** *21 (10), 1423-1427.*
5. *Li, Y.; Chooi, Y. H.; Sheng, Y.; Valentine, J. S.; Tang, Y., Comparative characterization of fungal anthracenone and naphthacenedione biosynthetic pathways reveals an α-hydroxylation-dependent Claisen-like cyclization catalyzed by a dimanganese thioesterase. J. Am. Chem. Soc.* ***2011,*** *133 (39), 15773-85.*
6. *Chiang, Y. M.; Szewczyk, E.; Davidson, A. D.; Entwistle, R.; Keller, N. P.; Wang, C. C.; Oakley, B. R., Characterization of the Aspergillus nidulans monodictyphenone gene cluster. Appl. Environ. Microbiol.* ***2010,*** *76 (7), 2067-74.*
7. *Schätzle, M. A.; Husain, S. M.; Ferlaino, S.; Müller, M., Tautomers of anthrahydroquinones: enzymatic reduction and implications for chrysophanol, monodictyphenone, and related xanthone biosyntheses. J. Am. Chem. Soc.* ***2012,*** *134 (36), 14742-5.*
8. *Han, Y. B.; Bai, W.; Ding, C. X.; Liang, J.; Wu, S. H.; Tan, R. X., Intertwined Biosynthesis of Skyrin and Rugulosin A Underlies the Formation of Cage-Structured Bisanthraquinones. J. Am. Chem. Soc.* ***2021,*** *143 (35), 14218-14226.*
9. *Griffiths, S.; Mesarich, C. H.; Saccomanno, B.; Vaisberg, A.; De Wit, P. J.; Cox, R.; Collemare, J., Elucidation of cladofulvin biosynthesis reveals a cytochrome P450 monooxygenase required for anthraquinone dimerization. Proc. Natl. Acad. Sci. U S A* ***2016,*** *113 (25), 6851-6.*
10. *Awakawa, T.; Yokota, K.; Funa, N.; Doi, F.; Mori, N.; Watanabe, H.; Horinouchi, S., Physically discrete beta-lactamase-type thioesterase catalyzes product release in atrochrysone synthesis by iterative type I polyketide synthase. Chem. Biol.* ***2009,*** *16 (6), 613-23.*
11. *Szwalbe, A. J.; Williams, K.; Song, Z.; de Mattos-Shipley, K.; Vincent, J. L.; Bailey, A. M.; Willis, C. L.; Cox, R. J.; Simpson, T. J., Characterisation of the biosynthetic pathway to agnestins A and B reveals the reductive route to chrysophanol in fungi. Chem. Sci.* ***2019,*** *10 (1), 233-238.*
12. *Szewczyk, E.; Chiang, Y. M.; Oakley, C. E.; Davidson, A. D.; Wang, C. C.; Oakley, B. R., Identification and characterization of the asperthecin gene cluster of Aspergillus nidulans. Appl. Environ. Microbiol.* ***2008,*** *74 (24), 7607-12.*
13. *Fujii, T.; Yamaoka, H.; Gomi, K.; Kitamoto, K.; Kumagai, C., Cloning and nucleotide sequence of the ribonuclease T1 gene (rntA) from Aspergillus oryzae and its expression in Saccharomyces cerevisiae and Aspergillus oryzae. Biosci. Biotechnol. Biochem.* ***1995,*** *59 (10), 1869-74.*
14. *Jin, F. J.; Maruyama, J.; Juvvadi, P. R.; Arioka, M.; Kitamoto, K., Development of a novel quadruple auxotrophic host transformation system by argB gene disruption using adeA gene and exploiting adenine auxotrophy in Aspergillus oryzae. FEMS Microbiol. Lett.* ***2004,*** *239 (1), 79-85.*
15. *Chen, L.; Liu, T.; Duan, Y.; Lu, X.; Yang, Q., Microbial Secondary Metabolite, Phlegmacin B1, as a Novel Inhibitor of Insect Chitinolytic Enzymes. J. Agric. Food. Chem.* ***2017,*** *65 (19), 3851-3857.*
